# Stress-resilient maize hybrids with stable agronomic performance: a pursuit to strengthen sustainable maize production in India

**DOI:** 10.3389/fpls.2025.1714259

**Published:** 2025-11-26

**Authors:** Ramesh Kumar, Yathish Rangappa Kondajji, Abhijit Kumar Das, Sushil Kumar, Yashmeet Kaur, Kamlesh Kumar, Jayasudha S., Chikkappa Gangadhar Karjagi, Manish B. Patel, Sunil Karad, B. S. Jat, Pervez H. Zaidi, Hanuman Sahay Jat

**Affiliations:** 1 Indian Council of Agricultural Research (ICAR)-Indian Institute of Maize Research, Ludhiana, Punjab, India; 2 Indian Council of Agricultural Research (ICAR)-Indian Institute of Maize Research Winter Nursery Centre, Hyderabad, India; 3 Banaras Hindu University, Varanasi, India; 4 Indian Council of Agricultural Research (ICAR) - Indian Institute of Maize Research (IIMR), Regional Maize Research & Seed Production Centre (RMR&SPC), Begusarai, India; 5 Anand Agricultural University, Godhra, India; 6 MPKV Regional Station, Kolhapur, India; 7 CIMMYT-Asia, Hyderabad, India

**Keywords:** mixed models, BLUE, BLUP, correlation, grain yield, principal component analysis, maize

## Abstract

Genotype by environment (G×E) interactions are of great interest for breeders to identify test locations and adapted genotypes. In the present study, 20 sub-tropical maize hybrids along with 5 commercial checks were planted at different locations under different ecologies (drought, high temperature, water logging and optimum environments) and evaluated for grain yield through the Best Linear Unbiased Estimations (BLUEs) and the Best Linear Unbiased Predictors (BLUPs). Genotypic and phenotypic correlations were also obtained among the different locations within the diverse ecologies. The trials were conducted during winter 2023 under drought, Spring 2024 under high temperature stress and under water logging during rainy 2023, respectively. The Genotype main effect plus genotype x environment interaction (GGE) biplot indicated that first and second principal components (PC1 and PC2) explained 100% of variation in drought, heat stress, water logging conditions. Under optimal conditions, it explained 75.81% variation. As per BLUE and BLUP, DKC 9144 and BH 417177 under drought, RCRMH 20 was under heat stress and BH 417144 under waterlogging were the best. Dendrogram was generated using Ward’s method of cluster analysis. Genotype RCRMH 20 was identified as the best performing genotype under heat (at locations Begusarai, Godhra and Kolhapur) and at water logging (Ludhiana, Hyderabad and Varanasi) with mean yield of 60.07 q/ha and 50.52 q/ha respectively. Based on these results it is recommended that hybrids namely MFH 2265, BH 417144, RCRMH 20 and BH 417177 may be tested in larger plot size before recommendation for release for commercial cultivation based on their performance in respective zones.

## 1 Introduction

Maize (*Zea mays* L.) is considered as an economically important crop in many Asian countries including India. It is an annual plant belonging to the grassassae family *Poaceae* that thrives well in various agroecologies because of its ability to adapt to multiple diverse environments (Acquaah, 2009; OECD, 2003). Also, it is the most crucial cereal globally, as 42% of the world’s human food calorie consumption comes from maize. Stable crop yields and high productivity ensure global food security. As per McKenzie and Williams (2015), global food production needs to increase by 60% or even double by 2050 to meet people’s food needs. This can be done by introducing new breeding lines and their testing in different environments for stability and potential for high yield.

Maize grain yields vary in different environments, so the selection of suitable genotypes in a range of conditions is essential for variety selection to enable improved site-specific planting. To increase yield potential even more, maize breeding efforts are focusing on producing high-yielding maize hybrids. The primary goal in the maize breeding program is to evaluate maize hybrids in various environments to identify high-yielding ones. The genotypes must be tested in a variety of environments across several ecological domains to identify and choose the most stable and adaptable genotypes.

Performance of most crop genotypes varies across the environments due to genotype–environment interactions. In multienvironment trials (METs), the effectiveness of variety selection is often constrained by substantial GEI effects, which leads to differential genotypic responses across the test sites and inconstant ranking patterns that complicate the identification of stable genotypes. So, it is essential to understand and explore the interactions caused by GEI effects on crop yield. Evaluation of yield potential and stability of the cultivars is crucial for assessing the value of promoting new varieties. In maize, GEIs are a key issue affecting floral traits like anthesis, silking and anthesis-silking interval (ASI), thousand kernel weight, grain yield, and breeding for quality traits (Ma et al., 2024).

Grain yield is affected significantly by adverse conditions such as drought, high-temperature stress, waterlogging, and combination of stresses. Since maize experiences acute water scarcity throughout the dry season, drought is recognized as the primary obstacle for reducing maize yields (Meng et al., 2016). Increasing temperatures have a negative impact on maize yield, whereas rainfall has a positive impact on maize production. For every 1^0^ rise in temperature, the yield of maize decreased by 1.6 units. For every 1-mm increase in rainfall, the yield increased by 0.05 units. An increase in temperature causes a decrease in pollination, thus leading to less grain filling and eventually a decline in crop yields (Soumya, 2022). As a result of the ongoing global warming, maize production at the global level is expected to be heavily impacted by droughts. Water scarcity, large and significant fluctuations in weather patterns, and the unpredictable nature of drought pose a serious threat to maize production worldwide. Among the environmental stresses, heat stress during the flowering period (HS) is the more demanding problem, which affects maize production, and the projections indicate a further decline in crop yields under increasing temperature. It has been reported that global maize yield potential may decrease to 45% by 2080s as compared with 1980s at extreme heat stress during anthesis (Cantarero et al., 1999). Prolonged anthesis-silking interval, reduction in kernel set, decreased photosynthesis rate, damaged cellular membrane, and decreased chlorophyll content have been reported under heat stress (Rattalino and Oteguia, 2013).

Climate change causing irregular and uneven rainfall for prolonged periods of time (Liu et al., 2021) increases the risk of waterlogging on maize plants. As a result of waterlogging, soil hypoxia occurs which reduces the amount of oxygen available to plants from the soil (Liu et al., 2020). Depending on the cultivar, crop growth stage, and duration and severity of stress, the amount of maize yield reduction caused by waterlogging might range from 7% to 80% (Tian et al., 2019). In the case of significant and major GEIs, maize hybrids with high adaptability to both optimal and waterlogging conditions and tolerant genotypes under waterlogging stress would be interesting to research upon. Most plant breeding programs aim to improve crop adaptability and resilience to biotic (pests and diseases) and abiotic (drought, salinity, and extreme temperatures) stresses, with an aim to achieve higher agricultural yields with minimal reduction (Chapman et al., 2012).

It is essential to evaluate the newly bred genotypes at several locations and years; thus, it makes multienvironment trials (MET) a prerequisite in crop improvement programs. The main purpose of METs is to capture the effect of diverse environmental parameters on the genotypic performance. Generally, significant genotype–environment interactions are often revealed by METs; thus, it makes selection of genotypes imprecise. If there is a significant genotype–environment interaction (GEI), then selection of entries is made for a particular location for specific adaptation.

Both the development of new cultivars and the deployment of newly released varieties require a selection among a larger set of candidate genotypes; so, estimation of genotypic values is at the heart of any breeding effort. Genetic factors can be considered random so they are usually included in the fixed-effects coincidence matrix. Therefore, it excludes the use of mixed models, which allow, for example, unbalanced data analysis (Henderson, 1975). Paraphrase it. The mixed model methodology has some advantages, such as predicting unrealized crosses (Gowda et al., 2013a), obtaining unbiased linear predictions, the best linear unbiased prediction (BLUP) for the random effects (genetic), and the best linear unbiased estimate (BLUE) for the fixed effects, associated with the estimated variance components for restricted maximum likelihood (REML). The use of this tool in breeding programs reduces time and costs by enabling the direct testing of the most promising crosses. Using BLUP as a selection tool and predictor of unknown crosses is very effective (Gowda et al., 2013b). Efficient selection of unknown crosses depends on an accurate understanding of the degree of the relatedness among the parents (Bernardo, 1995).

Variance analysis and multiple comparisons of yield facilitate the assessment of yield differences among varieties. However, the stability of varieties primarily arises from the genotype–environment interaction (G×E) effect. The conventional approaches for analysis of MET data are mainly ANOVA-based techniques such as AMMI (additive main effects and multiplicative interaction) and GGE (genotype and genotype-by-environment interaction) analysis. The GGE biplot is created by plotting the first two principal components (PC1 and PC2) produced from the environment-centered data’s singular value decomposition (Yan, 2001). The analysis of MET data in plant breeding is extremely challenging with conventional ANOVA-based methods possessing limitations as the complexity of MET data set increases. LMM models have important implications for improving accuracy and efficiency of MET data analysis, which is mandatory for increasing the genetic gains in plant breeding. Smith et al. (2001) employed the factor analytic multiplicative mixed (FAMM) models for G × E analysis. This method provides a good approximation as well as more robustness in general.

Understanding G×E interactions facilitates the identification of a stable genotype suitable for cultivation later. Therefore, in view of the above considerations, in the present study, the GGE biplot and the linear mixed models were used to analyze the yield of 25 maize hybrids under drought, heat stress, waterlogging, and irrigated environments to comprehensively evaluate the potentiality and stability of the test hybrids. The study was done with the objective to assess the differential performance of hybrids under different abiotic stresses, to ascertain the genotype–environment interaction for grain yield in the multienvironment trials, to estimate the significant differences within and across locations and to estimate the BLUEs and BLUPs for genotypes to identify the superior ones.

## 2 Materials and methods

### 2.1 Plant materials and experimental sites

Maize hybrid evaluation trials, comprising 25 hybrids (20 test and 5 check hybrid entries: CAH 1511, DKC 9144, P 3302, S 6668 Plus, and DKC 9178), were conducted under managed waterlogging, managed drought, and heat stress across different agroecological zones of India. The details of the experimental material are listed in **Table 1**. The 20 test hybrids are at the advanced stage of testing, whereas five checks are already released for cultivation under different agroclimatic zones under the All India Coordinated Research project (AICRP) system. The same set of hybrids was used for testing across different stresses and agroclimatic zones for generating information on their differential performance. The details of trials conducted during different planting seasons and locations along with latitude and longitude and dates of planting in 2023 and 2024 are given in **Table 2**.

**Table 1:** List of 25 maize hybrids and their contributing centers used in this study.

S. no.	Name	Contributing center
1.	OMH 22-4	OUAT, Bhubaneshwar
2.	AHD 2008	IARI, RRCD
3.	AHD 8751	IARI, RRCD
4.	JH 32662	Punjab Agricultural University, Ludhiana
5.	JH 32487	Punjab Agricultural University, Ludhiana
6.	AH 8323	IARI, RRCD
7.	IMH 9-222	ICAR-IIMR, Ludhiana
8.	JH 20088	Punjab Agricultural University, Ludhiana
9.	RCRMH 20	University of Agricultural Sciences, Raichur
10.	IMH 2-22K-4	ICAR-IIMR, Ludhiana
11.	BH 417189	Maize Research Centre, PJTSAU
12.	BH 417144	Maize Research Centre, PJTSAU
13.	AHD 2077	IARI, RRCD
14.	IMH 2-22K-7	ICAR-IIMR, Ludhiana
15.	IMH 2-22K-6	ICAR-IIMR, Ludhiana
16.	IMH 10-21K-2	Punjab Agricultural University, Ludhiana
17.	IMHSB 20K-10	ICAR-IIMR, Ludhiana
18.	BH 417177	Maize Research Centre, PJTSAU
19.	BH 417018	Maize Research Centre, PJTSAU
20.	MFH 2265	Tirhut College of Agriculture, Dholi
21.	Internal Check-1 (CAH 1511)	CIMMYT, Hyderabad
22.	Commercial Check-1 (DKC 9144)	Bayer Crop Science India Limited
23.	Commercial Check-2 (P 3302)	Corteva Agriscience
24.	Commercial Check-3 (S 6668 PLUS)	Syngenta
25.	Commercial Check-4 (DKC 9178)	Bayer Crop Science India Limited

**Table 2:** Details of the locations under different environments.

Environment	Season	Location	Dates of planting	Latitude	Longitude
Managed waterlogging	Rainy season in 2023	Ludhiana	19.07.2023	30\° 54\′ 32.47\″ N	75\° 51\′ 05.76 E
		Hyderabad	23.06.2023	17\° 23\′ 18.24\″ N	78\° 27\′ 59.04\″ E
		Varanasi	16.07.2023	25.3176\° N	82.9737\° E
Heat stress	Spring/summer season in 2024	Begusarai	27.03.2024	25.4182\° N	86.1272\° E
		Godhra	19.03.2024	22.7788\° N	73.6143\° E
		Kolhapur	21.03.2024	16.7064\° N	74.2482\° E
Managed drought	Winter season in 2023-24	Hyderabad	14.12.2023	17\° 23\′ 18.24\″ N	78\° 27\′ 59.04\″ E
		Godhra	2.12.2023	22.7788\° N	73.6143\° E
		Kolhapur	18.12.2023	16.7064\° N	74.2482\° E
Optimum	Rainy season in 2023	Ludhiana	15.07.2023	30\° 54\′ 32.47\″ N	75\° 51\’05.76 E
		Hyderabad	18.07.2023	17\° 23\′ 18.24\″ N	78\° 27\′ 59.04\″ E
		Varanasi	14.07.2023	25.3176\° N	82.9737\° E
		Begusarai	19.07.2023	25.4182\° N	86.1272\° E
		Godhra	15.07.2023	22.7788\° N	73.6143\° E
		Kolhapur	18.07.2023	16.7064\° N	74.2482\° E

### 2.2 Experimental design

The trials were conducted by following a randomized complete block design (RCBD) with two replications at all locations. Two seeds per hill of each test and check entries (treatments) were sown at the top of the hill in one row of 3 m with a spacing of 0.7 × 0.20 m. One seedling per hill was maintained by removing extra seedling at 15 days after germination to maintain the recommended plant population of 70,000/ha. The necessary agronomic and cultural practices were followed timely to ensure good plant growth and development till the imposition of stress conditions. The trials were conducted under optimal irrigated and different abiotic stress conditions like drought, heat, and waterlogging.

### 2.3 Managed drought, heat, and waterlogging stresses

In contrast to the optimum/optimal trial where all the necessary growing conditions were provided to ensure the full potential of genotype expression, specific stress conditions were created and imposed during a specific growth stage. The managed drought condition, low-moisture stress, was created during the flowering stage, the most-sensitive and critical stage of crop growth and development by withdrawing irrigation before the initiation of flowering till the completion of pollination. However, crop was grown under optimum growing conditions till the initiation of tasseling by providing irrigation for germination and also early vegetative growth. In addition, four rows as a protective border were included around the experimental area. The managed heat stress condition was created by taking up sowing deliberately in the second fortnight of March and coinciding the flowering period to high temperatures of April-May. The maximum temperature during flowering usually touched 40 °C to 45°C depending on the experimental sites. However, the temperature generally varied from 33 °C to 45°C during the whole cropping season, thus facilitating the screening under heat stress conditions. Since the flowering and grain filling stages coincided with high temperature in the April-May months with negligible incidence of rainfall and the relative humidity of 15%-46% subjected the hybrids to elevated temperature, it resulted in severe heat stress. The waterlogging stress condition was applied to the crop 35–40 days after sowing at the crop stage between knee high to pre-flowering (Liu et al., 2010; Zaidi et al., 2016). The waterlogging stress condition (continuous stagnation of water of 10 cm height for 7 days) was created by irrigating the trial area till the water stagnation followed by preventing leakage by making strong bunds around the entire experimental field. The development of anaerobic conditions at fields led to high waterlogging stress. The excess water was drained out after 7 days from the experimental field through surface drainage, and the crop was managed as per optimal practices of maize production.

### 2.4 Statistical analysis

Analysis of metric data from plant breeding and variety trials can usually be based on a linear mixed model (LMM) of the form


Y=X β+Z u+e

where Y is the vector of observations, β and *u* are the vectors of fixed and random effects, respectively, X and Z are the associated design matrices, and e is a random residual vector. The random effects are assumed to be distributed as *u*_∼_MVN**(0, G**) and *u*_∼_MVN **(0, R**), where MVN (µ, V) denotes the multivariate optimal distribution with mean vector µ and variance–covariance matrix V. The fixed effects can be estimated by best linear unbiased estimation (BLUEs), whereas random effects are estimated by best linear unbiased prediction (BLUPs). In practice, BLUE and BLUP need to be replaced by “empirical” BLUE and BLUP, respectively, meaning that variance components in G and R need to be replaced by their estimates, obtained preferably by restricted maximum likelihood (REML) (Patterson and Thompson, 1971). Both BLUE and BLUP may be computed by solving the mixed model equations (MME), given by Henderson (1986) and Searle et al. (1992).


$$\left[\begin{array}{cc} X'R^{-1}X & X'R^{-1}Z\\ Z'R^{-1}X & Z'R^{-1}Z+G^{-1} \end{array}][\begin{array}{c} \hat{\beta }\\ \hat{u} \end{array}]=[\begin{array}{c} X'R^{-1}y\\ Z'R^{-1}y \end{array}\right]$$

Often in variety testing and the development of new varieties, genotype effects are considered as fixed and thus become part of b in the mixed model. When genotypes can be treated as random, however, genotypic effects become part of u and thus are estimated by BLUP.

A GGE biplot was generated from the grain yield data collected from three experimental sites organized into a three-column data table of genotype–environment–yield, where each value represents the average yield of the corresponding genotype in the respective environment, known as the phenotype value (Yger). The linear statistical model for GGE biplot analysis is presented as follows:


$${\text{Y}}_{\text{ij}}-\text{μ}-{\text{β}}_{\text{j}}={\text{λ}}_{1}{\text{ξ}}_{\text{i}1}{\text{η}}_{\text{j}1}+{\text{λ}}_{2}{\text{ξ}}_{\text{i}2}{\text{η}}_{\text{j}2}+{\text{ϵ}}_{\text{ij}}$$

where Υij is the measured mean (DBH) of genotype i in environment j, μ is the overall mean, βj is the main effect of environment j, μ + βj is the mean yield across all genotypes in environment j, λ1 and λ2 are the singular values (SV) for the first and second principal components (PC1 and PC2), respectively, ξ i1 and ξ i2 are the eigenvectors of genotype i for PC1 and PC2, respectively, ηj1 and ηj2 are the eigenvectors of environment j for PC1 and PC2, respectively, and ϵij is the residual associated with genotype i in environment j.

The genotypic and environmental components of variance for grain yield were estimated from the ANOVA. Cluster analysis was done using Ward’s method, and a dendrogram was generated. The BLUP, BLUE, correlation coefficients, and heritability were provided by the PROC MIXED and PROC COR function, respectively, of SAS 9.3 (SAS Institute, Cary, North Carolina, USA).

## 3 Results

### 3.1 Analysis of variance using linear mixed models

As four different ecologies (drought, heat stress, waterlogging, and optimum) were considered for the study, within-environment ANOVA was carried out for grain yield using linear mixed models BLUE and BLUP in this study (**Table 3**). As per BLUP, under drought conditions, there were significant differences among tested environments but genotypes and G × E interactions were non-significant. The significant environment effect indicates that water deficit conditions across the three locations played a significant role in determining yield outcomes. For heat stress, the G × E interaction as well as the environments showed significant differences, but genotypes were non-significant. The significant G × E interaction indicated that genotypic performance varied across environments, and there is evidence of a crossover interaction. For waterlogging, significant differences among the genotypes were observed, which proved that the hybrids used in this study had significant differences in grain yield. However, the G × E interaction as well as the environments were non-significant under waterlogging. Under optimum conditions, all three sources were significant.

**Table 3:** Variance analysis for 25 maize genotypes for yield components by LMM model.

S. no.	Statistic	Drought		Heat		Waterlogging		Optimum	
		BLUP_GY	BLUE_GY	BLUP_GY	BLUE_GY	BLUP_GY	BLUE_GY	BLUP_GY	BLUE_GY
Variance components and genetic parameters									
1	Heritability	0.393	NA	0.380	NA	0.659	NA	0.87	NA
2	Genotype variance	8.554	NA	22.85	NA	49.265	NA	161.44	NA
3	Gen×Loc variance	1.787	NA	81.82	NA	9.015	NA	127.29	NA
4	Environment variance	1,099.9	NA	233.47	NA	12.42	NA	34.249	NA
5	Residual variance	75.372	75.372	59.57	59.57	134.79	134.79	18.11	18.11
6	Grand mean	40.296	40.296	47.190	47.19	36.57	36.57	87.54	87.54
7	LSD	6.5575	10.314	10.825	17.34	11.92	14.35	13.28	13.34
8	CV	21.544	21.544	16.356	16.35	31.74	31.74	4.86	4.86
Random effects									
9	Replicates	2	2	2	2	2	2	2	2
10	Environments	3	3	3	3	3	3	6	6
11	Genotype significance	0.1470^ns^	0.040*	0.166 ^ns^	0.0477*	0.0015*	5.31E−05*	2.99E−15*	0.000*
12	Gen×Env significance	0.8602 ^ns^	0.860 ^ns^	4.29E−07*	4.29E−07*	0.63 ^ns^	0.63 ^ns^	5.79E−46*	5.79E−46*
13	Env significance	0.0004*	0.000*	0.0153*	0.0153*	0.32 ^ns^	0.32 ^ns^	0.015*	0.015*

*Significant at the 0.05 probability levels; ns: not significant at p = 0.05.

As per the BLUE by LMM, under drought ecology, both the genotypes and environments had significant variation whereas the G × E interaction was non-significant. Under HS, all three components were significant. Thus, these results under heat stress (HS) indicate that there is variability between individuals, environments were contrasting, and the selection made considering both genotypes and environments together will provide gains generated from contrasting environments with the genotypes becoming more adapted and stable. For waterlogging, only genotypic effects were significant whereas the G × E interaction as well as the environments were non-significant. Under optimum conditions, all three sources were significant. The significant G × E interaction implied how different genotypes respond to varying environmental conditions. This interaction suggests that some genotypes perform better in specific environments, and understanding these interactions can aid in recommending the best genotype for specific conditions.

Later, a combined ANOVA over all the tested environments was performed to check the significance of GEI for grain yield (**Table 4**). Genotypes exhibited a significant difference for the grain yield, indicating differences in means of genotypes, thus making selection amenable. Significant differences among the environments for grain yield implied an environmental mean difference for the traits and its influence in expression of these traits. The significant G × E interaction suggests that there is evidence of a crossover interaction among genotypes and environments. The significant G × E interactions also indicates that maize genotypes exhibited diverse mean grain yield performances across E1, E2, and E3 environments, implying a strong likelihood of identifying a genotype with both specific and broad adaptability. Environmental variances constituted a major proportion of variances as contrary to genotypic, residual and genotype × location variances under drought (1099.9) and heat (233.47) (HS) conditions, whereas residual and genotypic variances form a higher part under waterlogging (49.26) and optimum (161.4) conditions.

**Table 4:** Variance analysis by LMM model across all environments.

S. no.	Statistic	BLUP_GY	BLUE_GY
1	Heritability	0.76	NA
2	Genotype variance	32.07	NA
3	Gen × Loc variance	118.08	NA
4	Environment variance	762.18	NA
5	Residual variance	61.19	61.19
6	Grand mean	59.83	59.83
7	LSD	8.12	8.75
8	CV	13.07	13.07
9	Replicates	2	2
10	Environments	15	15
11	Genotype significance	2.33E−09*	1.15E−10*
12	Gen × Env significance	1.3E−45*	1.3E−45*
14	Environment significance	1.07E−13*	1.07E−13*

*Significant at the 0.05 probability levels.

### 3.2 Mean performance of test hybrids under different stresses

The trials for drought were conducted at Anand Agricultural University (Godhra: E1), MPKV Regional Station (Kolhapur: E2), and CIMMYT-Asia (Hyderabad: E3) during Winter 2023. At E1, the maximum temperatures (Tmax) and relative humidity (%) during the months of February and March 2024 were 31.3°C, 91.2 °C, and 37.7°C and 72%, respectively. The total rainfall (mm) recorded was 6 and 0 mm during the flowering period in February and March, respectively, which indicated the severity of stress imposed on plants. At E2, the maximum temperature and RH were 36.5°C and 96% during the February 2024, respectively. There was no rainfall during the flowering period. At E3, the maximum temperature and RH were 35.2°C and 94% during February 2024, respectively. There was no occurrence of rainfall during the flowering period. The rainfall, mean temperature, and mean relative humidity of E1, E2, and E3 are shown in **Supplementary Figures 1A-C**, respectively.

The grain yield ranged from 22.14 to 62.14 q/ha, 64.13 to 87.13 q/ha, and 0.14 to 24.29 q/ha with mean grain yields of 37.20, 74.96, and 8.73 q/ha at E1, E2, and E3, respectively. The overall mean grain yield at all three locations was 40.29 q/ha. At E1, the highest yield was of commercial check-1 (DKC 9144: 62.14 q/ha). No genotype outyielded the check at this location. However, BH 417177, JH 32487, and RCRMH 20 had high grain yields of 52.14, 50.64, and 42.14 q/ha, respectively. At E2, genotypes IMH 22K-6, BH 417189, and IMH 9–222 performed best with grain yields of 87.13, 86.64, and 83.48 q/ha, which is more than the commercial check-4 (DKC 9178; 83.46 q/ha). Genotype MFH 2265 was at par with the check with grain yield of 82.51 q/ha. At E3, the highest grain yield (24.29 q/ha) was recorded by commercial check-1 (DKC 9144). However, MFH 2265, IMH 2-22K-4, BH 417144, and IMHSB 20K-10 also performed well with grain yields of 16.28, 14.71, 13.43, and 12.71 q/ha, respectively (**Table 5**).

**Table 5:** Mean grain yield (q/ha) of 25 maize hybrids in different locations under tested stress environments.

S.n.	Hybrid	Waterlogging			Heat			Drought			Optimum					
		E1	E2	E3	E1	E2	E3	E1	E2	E3	E1	E2	E3	E4	E5	E6
1	OMH 22-4	21.21	21.57	31.43	37.14	48.43	49.92	33.57	75.53	4.28	52.51	81.54	79.71	70.80	58.38	83.53
2	AHD 2008	29.93	26.43	35.71	44.29	44.43	55.57	32.86	67.91	6.28	66.48	81.24	76.30	77.55	76.76	66.08
3	AHD 8751	27.57	31.86	37.14	40.71	6.71	42.45	23.29	74.60	9.57	70.33	88.81	81.45	77.10	73.82	75.64
4	JH 32662	30.36	18.29	41.43	28.57	54.14	65.86	30.71	64.13	5.00	60.55	79.72	75.86	82.63	78.06	78.44
5	JH 32487	31.36	25.00	42.86	39.29	43.57	82.66	50.64	75.63	9.29	73.88	89.31	94.80	77.86	73.26	82.85
6	AH 8323	25.79	13.14	23.57	37.14	40.43	79.30	31.43	77.38	3.00	65.60	107.12	97.83	82.37	84.64	85.21
7	IMH 9-222	37.29	45.00	46.43	27.86	31.43	55.87	40.64	83.48	11.57	60.66	67.05	94.49	80.13	70.79	60.05
8	JH 20088	46.64	35.14	61.43	33.57	48.14	69.60	22.14	77.30	6.14	90.29	87.22	93.72	64.61	93.39	88.49
9	RCRMH 20	47.57	52.57	51.43	42.14	45.00	93.06	42.14	68.44	3.43	60.11	94.85	101.32	85.08	67.54	98.66
10	IMH 2-22K-4	47.79	33.29	57.14	50.00	52.14	55.43	33.57	72.31	14.71	89.49	82.85	95.53	78.15	85.84	91.73
11	BH 417189	39.14	45.29	38.57	40.00	36.86	58.77	30.00	86.64	10.71	61.34	111.88	90.15	79.34	62.40	91.47
12	BH 417144	61.93	56.14	39.29	55.71	43.14	55.85	33.57	76.36	13.43	69.84	99.63	93.16	81.62	70.77	81.23
13	AHD 2077	34.86	27.14	22.14	12.86	27.43	50.04	27.86	69.17	5.00	80.10	87.34	96.41	70.39	88.81	63.99
14	IMH 2-22K-7	34.57	19.00	65.71	35.00	50.29	65.23	32.07	75.00	10.14	100.15	86.26	81.82	79.60	102.34	68.41
15	IMH 2-22K-6	51.07	35.14	30.71	50.00	33.14	62.90	41.43	87.13	8.57	83.44	98.78	102.50	74.79	84.10	98.45
16	IMH 10-21K-2	29.36	27.86	22.86	15.71	39.57	66.32	37.14	72.51	0.14	56.22	49.39	98.49	78.78	58.25	98.64
17	IMHSB 20K-10	20.25	37.86	41.43	35.71	34.86	91.10	40.71	68.91	12.71	50.54	64.65	104.68	76.21	63.28	86.04
18	BH 417177	40.50	48.00	50.00	40.71	42.00	86.68	52.14	75.13	12.64	81.03	81.52	94.46	80.99	82.55	122.61
19	BH 417018	32.36	41.71	50.71	40.00	53.43	57.69	40.00	68.43	6.71	75.75	71.25	106.56	73.68	74.28	76.36
20	MFH 2265	31.29	41.00	42.85	47.86	31.29	72.62	32.85	82.51	16.28	114.70	67.73	88.08	91.27	84.08	115.01
21	CAH 1511 (check)	45.93	41.71	45.71	48.57	42.86	64.57	42.71	72.85	4.14	94.87	110.50	104.47	90.97	100.80	121.09
22	DKC 9144 (check)	34.36	27.29	40.00	26.43	30.86	67.65	62.14	72.83	24.29	109.35	114.37	112.73	91.98	107.80	124.61
23	P 3302 (check)	26.36	4.43	40.71	47.86	38.86	65.83	42.14	72.70	12.43	113.22	119.09	106.09	98.54	110.17	125.20
24	S 6668 Plus (check)	38.21	28.86	27.86	17.14	42.29	58.85	40.71	83.46	5.00	117.36	115.18	111.65	97.84	112.88	124.52
25	DKC 9178 (check)	37.21	32.15	37.14	23.21	31.29	55.36	33.57	73.65	2.71	122.99	121.31	116.56	95.13	113.87	129.52

High-temperature stress screening was done at ICAR-IIMR RMR&SPC (Begusarai: E1), Anand Agricultural University (Godhra: E2), during spring 2024 and MPKV Regional Station (Kolhapur; E3) during summer 2024. At Begusarai (E1), during the flowering period in May and June (2024), the observed maximum temperatures (°C) were 37.6 and 37.1, relative humidities (%) were 92 and 87, and total rainfall (mm) were 87.4 and 94.8, respectively. At Godhra (E2), maximum temperatures of 42.7°C and 39.7°C, relative humidities of 84.2% and 96.5%, and rainfall of 1.8 and 23.4 mm were recorded in May and June 2024, respectively. At E3, the maximum temperature and RH were 41°C and 96% in month of May 2024, respectively; however, a negligible rainfall of 4.13 mm was recorded. The calculated GDDs {(growing degree days, i.e., (Tmax-Tmin/2-Base temperature (8°C)} were 0, 1.3, and 2.75 under E1, E2, and E3, respectively. High temperatures lead to high number of GDD; also, temperatures above 32°C damage the pollen and reduce grain filling. The rainfall, mean temperature, and mean relative humidity of E1, E2, and E3 are shown in **Supplementary Figure 2A**, **1A, B**, respectively.

The grain yields at E1, E2, and E3 ranged from 12.86 to 55.71 q/ha, 6.71 to 54.14 q/ha, and 42.45 to 93.06 q/ha, respectively. The mean grain yields observed at all three locations were 36.70, 39.70, and 65.71 q/ha with an overall location mean of 47.19 q/ha. Genotypes BH 417144, IMH 2-22K-4, and IMH 22K-6 had grain yields of 55.71, 50.0, and 50.0 q/ha, which performed better than the check commercial check-1 (DKC 9144; 48.57 q/ha) at E1. At E2, genotypes JH 32662, BH 417018, IMH 2-22K-4, IMH 22K-7, and OMH 22–4 were among the best-performing ones with grain yields of 54.14, 53.43, 52.14, 50.29, and 48.43 q/ha. At the third location, genotypes RCRMH 20, IMHSB 20K-10, BH 417177, JH 32487, and AH 8323 were the highest yielders with yields of 93.06, 91.10, 86.68, 82.66, and 79.30 q/ha, respectively.

Trials for waterlogging were implemented at the ICAR-Indian Institute of Maize Research (Ludhiana: E1), CIMMYT-Asia (Hyderabad: E2), and Banaras Hindu University (Varanasi: E3) during Rainy 2023. At E1 (Ludhiana), during months of August and September, 2024, the Tmax were 32.7°C and 33.3°C and RH were 92.5 and 96, respectively. A total heavy rainfall of 100.9 mm was also observed, which indicated the cumulative impact of managed waterlogging stress and increased rainfall. As a result, the soil moisture levels became high (they exceed field capacity) and absence of air-filled pores caused the starvation of oxygen in plant roots. The erratic and uneven distribution of rainfall during the flowering period led to the effect of relatively much more severe effects of waterlogging. At E2 (Hyderabad), 33.2°C and 31.2°C (Tmax) and 95 and 98 (RH) were recorded during August and September 2024. A negligible rainfall of 4.6 mm was also recorded. At E3 (Varanasi), 130.8 mm rainfall, 34.6°C maximum temperature, and 95% RH were recorded during September 2024. The rainfall, mean temperature, and mean relative humidity of E1, E2, and E3 are shown in **Supplementary Figure 3A**, **1C, B**, respectively.

The grain yield ranged from 20.25 to 61.93 q/ha, 4.43 to 56.14 q/ha, and 22.14 to 65.71 q/ha with mean yields of 36.12, 32.63, and 40.97 q/ha at E1, E2, and E3, respectively. At E1, genotypes BH 417144, IMH 22K-6, IMH 2-22K-4, RCRMH 20, and JH 20088 showed exceptional high yields of 61.9, 51.07, 47.79, 47.57, and 46.64 q/ha respectively. Genotypes BH 417144, RCRMH 20, BH 417177, BH 417189, and IMH 9–222 had mean yields of 56.14, 52.57, 48.00, 45.29, and 45.00 q/ha, respectively. At E3, genotypes IMH 22K-7, JH 20088, IMH 2-22K-4, RCRMH 20, and BH 417018 had grain yields of 65.71, 61.43, 57.14, 51.43, and 50.71 q/ha, respectively.

Optimal/optimum trials were conducted at all the locations, *viz*., ICAR-IIMR RMR&SPC (Begusarai: E1), Anand Agricultural University (Godhra: E2), MPKV Regional Station (Kolhapur: E3), ICAR-Indian Institute of Maize Research (Ludhiana: E4), Banaras Hindu University (Varanasi: E5), and CIMMYT-Asia (Hyderabad: E6). The grain yields of E1, E2, E3, E4, E5, and E6 ranged from 50.5 to 122.9 q/ha, 49.3 to 121.3 q/ha, 75.8 to 116.5 q/ha, 64.61 to 98.5 q/ha, 58.2 to 113.8 q/ha, and 60.0 to 129.5 q/ha with mean yields of 80.8, 90.3, 95.9, 81.4, 83.1, and 93.5 q/ha, respectively. The overall location mean grain yield was 87.5 q/ha.

At all the six locations, none of the genotypes surpassed all the checks in terms of grain yield. However, some genotypes performed relatively good or at par. At E1, commercial check-4 (DKC 9178) and commercial check-3 (S 6668 Plus) had the highest grain yields of 122.9 and 117.3 q/ha. Genotype MFH 2265 had a grain yield of 114.7 q/ha at E1. At second location E2, commercial check-4 (DKC 9178), commercial check-2 (P 3302), commercial check-3 (S 6668 Plus), and commercial check-1 (DKC 9144) were the top performing with 121.3, 119.0, 115.1, and 114.3 q/ha yield, respectively. Genotype BH 417189 was identified as the good hybrid with a yield of 111.8 q/ha at E2. At E3, commercial check-4 (DKC 9178), commercial check-1 (DKC 9144), and commercial check-3 (S 6668 Plus) were best performers with 116.5, 112.7, and 111.6 q/ha. Genotype BH 417018 was at the fourth rank with a grain yield of 106.5 q/ha.

At E4, commercial check-2 (P 3302), commercial check-3 (S 6668 Plus), commercial check-4 (DKC 9178), and commercial check-1 (DKC 9144) were the best performing with 98.5, 97.8, 95.1, and 91.9 q/ha. Genotype MFH 2265 emerged as relatively good with a 91.2-q/ha grain yield. At E5, genotypes commercial check-4 (DKC 9178), commercial check-3 (S 6668 Plus), commercial check-2 (P 3302), and commercial check-1 (DKC 9144) performed best with 113.8, 112.8, 110.1, and 107.8 q/ha yield. Genotype IMH 22K-7 was good with yield 102.3 q/ha. At E6, genotypes commercial check-4 (DKC 9178), commercial check-2 (P 3302), commercial check-1 (DKC 9144), and commercial check-3 (S 6668 Plus) were good with 129.5, 125.2, 124.6, and 124.5 q/ha. Genotype BH 417177 had 122.6 q/ha grain yield potential.

To conclude, for the genotypic performance under optimal conditions in E1, E2, E3, E4, E5, and E6, genotypes MFH 2265 (114.705, 67.73, 88.08, 91.27, 84.08, and 115.01 q/ha), JH 20088 (90.29, 87.22, 93.72, 64.61, 93.39, and 88.49 q/ha), and IMH 22K-7 (100.15, 86.26, 81.82, 79.60, 102.34, and 68.41 q/ha) were identified as the overall potential hybrids.

### 3.3 Estimation of BLUE and BLUP values

Best linear unbiased predictors (BLUP) and BLUE values were estimated for all genotypes studied for varying locations; thus, it was possible to identify DKC 9144 and BH 417177 as the best hybrid combinations under drought ecologies. RCRMH 20 was identified as the best one under heat stress. For waterlogging, BH 417144 was adjudged as the best hybrid whereas under optimum, none of the hybrids outyielded the checks; however, MFH 2265 was at par with the checks (**Table 6**). Hence, these identified hybrids can be recommended for large-scale testing over locations. According to Bernardo (1995), BLUP can effectively predict when the degree of relationship between parents is known. Guo et al. (2013) corroborated this when they predicted hybrid corn from inbred lines and concluded that genetic architecture determines the accuracy of prediction.

**Table 6:** Estimated BLUP and BLUE under diverse environments.

S. no.	Genotype	Drought		Heat		Waterlogging		Optimum		
		BLUP_GY	BLUE_GY	BLUP_GY	BLUE_GY	BLUP_GY	BLUE_GY	BLUP_GY		BLUE_GY
1	AH8323	39.10	37.27	49.13	52.29	26.19	20.83	87.17		87.12
2	AHD2008	38.47	35.68	47.53	48.09	32.69	30.69	75.73		74.06
3	AHD2077	37.81	34.01	40.69	30.11	30.95	28.05	81.95		81.17
4	AHD8751	38.53	35.81	40.63	29.95	33.68	32.19	79.05		77.85
5	BH417018	39.54	38.38	48.40	50.37	39.88	41.59	80.62		79.64
6	BH417144	40.62	41.11	48.85	51.57	47.04	52.45	83.30		82.70
7	BH417177	42.79	46.63	50.71	56.46	42.89	46.16	90.15		90.52
8	BH417189	41.14	42.45	46.43	45.21	39.49	40.99	83.35		82.76
9	CAH1511	40.14	39.90	49.02	52.0	41.76	44.45	101.78		103.78
10	DKC9144	45.33	53.08	45.08	41.64	34.79	33.88	107.35		110.14
11	DKC9178	38.85	36.64	43.16	36.62	35.86	35.50	112.98		116.56
`12	IMH10-21K-2	38.83	36.59	44.65	40.53	30.05	26.68	75.05		73.29
13	IMH2-22K-4	40.25	40.19	49.22	52.52	42.83	46.07	87.29		87.26
14	IMH2-22K-6	42.42	45.70	47.75	48.68	38.15	38.97	89.99		90.34
15	IMH2-22K-7	39.81	39.07	48.32	50.17	38.67	39.76	86.56		86.42
16	IMH9-222	42.24	45.23	43.84	38.38	40.74	42.90	74.08		72.19
17	IMHSB20K-10	40.48	40.78	49.73	53.89	34.33	33.17	75.87		74.23
18	JH20088	38.28	35.19	48.42	50.43	43.93	47.74	86.44		86.28
19	JH32487	42.22	45.18	50.22	55.17	34.26	33.07	82.67		81.99
20	JH32662	37.53	33.28	48.07	49.52	32.25	30.02	77.31		75.87
21	MFH2265	41.70	43.88	48.48	50.58	37.76	38.37	92.74		93.47
22	OMH22-4	39.30	37.79	46.41	45.16	28.77	24.73	73.10		71.07
23	P3302	41.13	42.42	48.58	50.84	28.17	23.83	109.02		112.05
24	RCRMH20	39.39	38.00	52.08	60.06	45.76	50.52	84.95		84.59
25	S6668PLUS	41.38	43.05	44.23	39.42	33.32	31.64	110.07		113.24

### 3.4 Genotypic and phenotypic correlations

Phenotypic correlation refers to the observable correlation between two characters and denoted as rP. Genotypic correlation refers to the heritable association between two characters and designated as rG. Under drought conditions, Kolhapur had a negative genotypic (−0.03) as well as phenotypic correlation (−0.007) with Godhra. Hyderabad had a positive genotypic and phenotypic correlation with Godhra (0.99, 0.42) and Kolhapur (0.99, 0.20). Under HS, the genotypic as well as phenotypic correlation among Godhra and Begusarai was positive (0.228 and 0.155). Kolhapur also had a positive genotypic as well as phenotypic correlation with Begusarai (0.151, 0.32) and Godhra (0.32, 0.24). For waterlogging, Hyderabad had a positive genotypic as well as phenotypic correlation (0.99 and 0.60) with Ludhiana. Varanasi had a positive genotypic as well as phenotypic correlation with Ludhiana (0.70 and 0.25) and Hyderabad (0.42 and 0.18) (**Table 7**). Under optimum ecology, all the environments (Godhra, Kolhapur, Begusarai, Ludhiana, Varanasi, and Hyderabad) had a positive genotypic as well as phenotypic correlation among themselves (**Table 8**). The genotypic correlations between Godhra and Hyderabad, and Hyderabad and Kolhapur under drought, and between Hyderabad and Ludhiana under waterlogging were statistically significant. The phenotypic correlations between Hyderabad and Godhra, and Hyderabad and Ludhiana, were observed significant. As values of rG are higher than rP between the environments, thus there is genetically a strong association between two environments. As the value is zero (between Kolhapur and Godhra) or non-significant, these two environments are independent.

**Table 7:** Genotypic and phenotypic correlations among the stressed locations in diverse ecologies.

Drought	Genotypic correlation		Phenotypic correlation	
	Godhra	Kolhapur	Godhra	Kolhapur
Kolhapur	−0.03^ns^		−0.00 ^ns^	
Hyderabad	0.99*	0.99*	0.42*	0.20 ^ns^
Heat				
	Begusarai	Godhra	Begusarai	Godhra
Godhra	0.22 ^ns^		0.15 ^ns^	
Kolhapur	0.15 ^ns^	0.32 ^ns^	0.12 ^ns^	0.24 ^ns^
Waterlogging				
	Ludhiana	Hyderabad	Ludhiana	Hyderabad
Hyderabad	0.99*		0.60*	
Varanasi	0.70 ^ns^	0.42 ^ns^	0.25 ^ns^	0.18 ^ns^

*Significant at the 0.05 probability levels, ns: non-significant at the 0.05 probability levels.

**Table 8:** Genotypic and phenotypic correlations among different locations under optimum crop growth regimes.

Environment	Genotypic correlation				
	Begusarai	Godhra	Kolhapur	Ludhiana	Varanasi
Godhra	0.59				
Kolhapur	0.51	0.46			
Ludhiana	0.69	0.59	0.52		
Varanasi	0.94*	0.67	0.50	0.63	
Hyderabad	0.67	0.54	0.67	0.78	0.56
	Phenotypic correlation				
Godhra	0.57*				
Kolhapur	0.48*	0.43*			
Ludhiana	0.64*	0.55*	0.47*		
Varanasi	0.92*	0.65*	0.48*	0.60*	
Hyderabad	0.65*	0.53*	0.63*	0.74*	0.56*

*Significant at the 0.05 probability levels.

### 3.5 Correlations of different environments with principal components

The correlation between the distinct environments and the principal component is shown in **Table 9**. Under drought, Godhra had a positive correlation with PC1 and PC3 (1 and 1) whereas it had a negative correlation (−0.49) with PC2. Kolhapur had a negative correlation with PC1 and PC2 (−1 and −0.49) but a positive correlation with PC3 (1). Hyderabad had a positive correlation with PC1 (7.99), whereas it had a negative correlation with PC2 and PC3 (−1 and −0.09). Under HS, Begusarai had a positive correlation with PC1 (1) whereas it had a negative correlation with PC2 and PC3 (−0.10 and −1). Godhra had a negative correlation with PC1 and PC3 (−0.44 and −0.71) whereas it had a positive correlation with PC2 (1). Kolhapur had a negative correlation with all the principal components PC1, PC2, and PC3 (−0.78, −0.70, and −0.87). Under waterlogging conditions, Ludhiana had a positive correlation with PC1 (0.99) whereas it had a negative correlation with both PC2 and PC3 (−0.47 and −0.99). Hyderabad had a positive correlation with PC1 and PC3 (1 and 1), whereas it was negatively correlated with PC2 (−0.47). Varanasi had a negative correlation with PC1 and PC2 (−0.95 and −1), whereas it had a positive correlation with PC3 (0.0009).

**Table 9:** Correlations of principal components (PC1, PC2, and PC3) with different environments.

Drought	Godhra	Kolhapur	Hyderabad			
PC1	1	−1	7.99E−13			
PC2	−0.49	−0.49	−1			
PC3	1	2	−0.99			
Heat	Begusarai	Godhra	Kolhapur			
PC1	1	−0.44	−0.78			
PC2	−0.10	1	−0.70			
PC3	−1	−0.71	−0.87			
Waterlogging	Ludhiana	Hyderabad	Varanasi			
PC1	0.99	1	−0.95			
PC2	−0.47	−0.47	−1			
PC3	−0.99	1	0.0009			
Optimum	Begusarai	Godhra	Kolhapur	Ludhiana	Varanasi	Hyderabad
PC1	0.84	0.62	−0.89	−0.085	1	−0.68
PC2	−0.51	0.51	0.36	−1	−0.18	−0.78
PC3	0.664	−1	0.70	−0.60	0.55	−0.13
PC4	0.221	0.378	−0.16	−0.77	0.043	1
PC5	0.836	0.002	−0.13	−0.10	−1	−0.24
PC6	−0.42	−0.79	−1	−0.44	−0.15	−0.10

Under optimum crop growth conditions, Begusarai had a positive correlation with PC1 (0.84), PC3 (0.66), PC4 (0.22), and PC5 (0.83) whereas it had a negative correlation with PC2 (−0.51) and PC6 (−0.42). Godhra had a positive correlation with PC1, PC2, PC4, and PC5 (0.62, 0.51, 0.37, and 0.002, respectively), whereas it was negatively correlated with PC3 and PC6 (−1 and −0.79). Kolhapur was positively correlated with PC2 and PC3 (0.36 and 0.70), whereas it was negatively correlated with all other principal components PC1, PC4, PC5, and PC6 (−0.89, −0.16, −0.13, and −1). Ludhiana was negatively correlated (−0.08, −1, −0.61, −0.77, −0.10, and −0.44) with all principal components (PC1, PC2, PC3, PC4, PC5, and PC6). Varanasi was positively correlated with PC1 (1), PC3 (0.55), and PC4 (0.043), whereas it was negatively correlated with PC2 (−0.18), PC5 (−1), and PC6 (−0.15). Hyderabad had a negative correlation with PC1 (−0.68), PC2 (−0.78), PC3 (−0.13), PC5 (−0.24), and PC6 (−0.10), whereas it was positively correlated with PC5 (−0.24).

### 3.6 Cluster analysis

**Figure 1:**
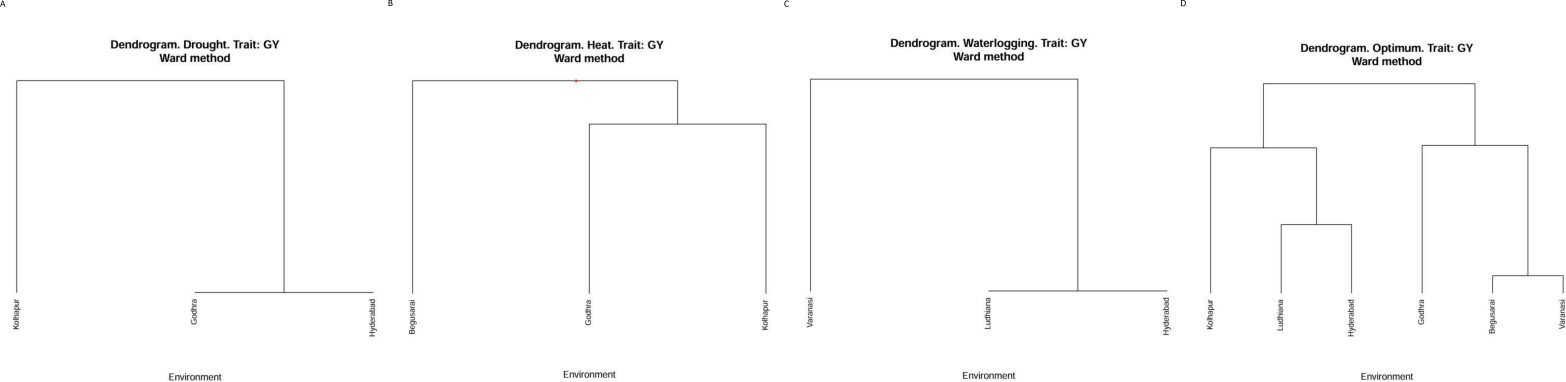
**(A)** Cluster analysis for drought ecology. **(B)** Cluster analysis for heat stress ecology. **(C)** Cluster analysis for waterlogging ecology. **(D)** Cluster analysis for optimum crop growth conditions.

Cluster analysis was used (Ward’s method) based on grain yield in the different locations within the diverse ecologies to classify different environments into similar classes. As it appears in **Figure 1A**, the three locations under drought ecology were classified in two groups. Kolhapur falls under one group, whereas Godhra and Hyderabad fall in another one. Considering HS (**Figure 1B**), Begusarai was categorized into one group. Godhra and Kolhapur were in another group under HS. Under waterlogging conditions (**Figure 1C**), Varanasi falls under one group and another group has Ludhiana as well as Hyderabad. Under optimum conditions (**Figure 1D**), Kolhapur, Ludhiana, and Hyderabad fall in one class. Another one was constituted by Godhra, Begusarai, and Varanasi. Classifying the locations according to the grain yield of genotypes with sophisticated multivariate techniques can reduce the cost of time and money in crop improvement.

### 3.7 GGE biplot

**Figure 2:**
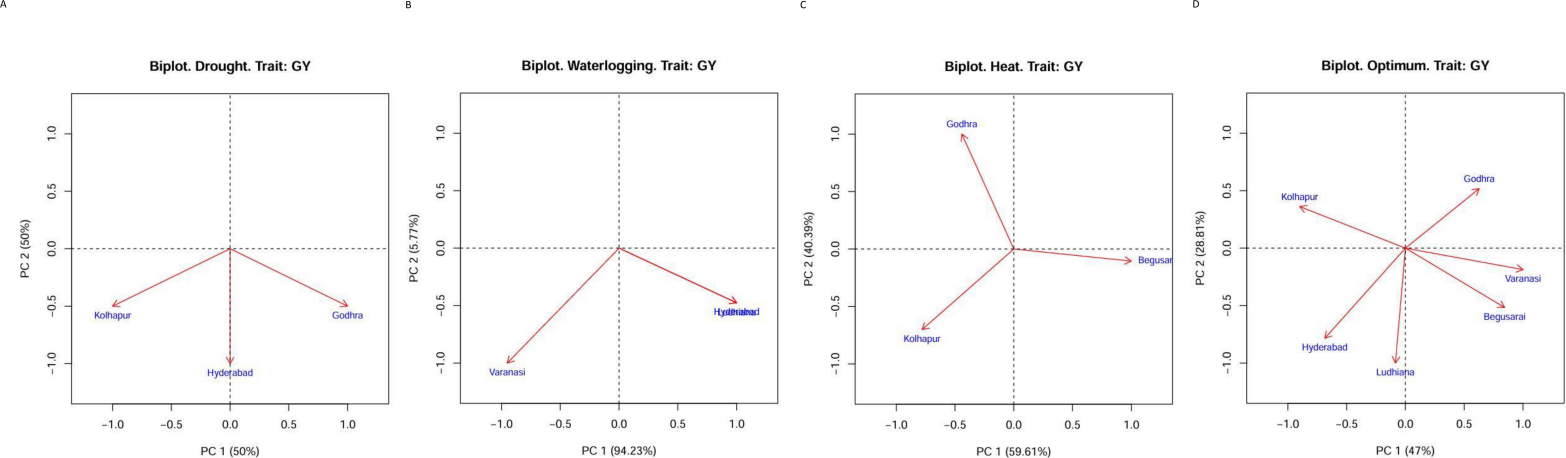
**(A)** GGE biplot for drought conditions. **(B)** GGE biplot for heat conditions. **(C)** GGE biplot for waterlogging conditions. **(D)** GGE biplot for optimum conditions.

The GGE biplot indicated that first and second principal components (PC1 and PC2) explained 100% of variation in drought (PC1: 50.0, PC2: 50.0), heat stress (PC1: 59.61, PC2: 40.39), and water logging (PC1: 94.23, PC2: 5.77) conditions. Under optimal conditions, AMMI explained 75.81% variation with PC1: 47 and PC2: 28.81 (**Figures 2A-D**). The lines that connect the test environments to the biplot origin are called environment vectors. The cosine of the angle between the vectors of two environments approximates the correlation between them. As per **Figures 2A-D**, under drought conditions, all three locations were positively correlated (an acute angle). For HS, Kolhapur and Godhra were not correlated (a right angle) and Kolhapur and Begusarai were slightly negatively correlated (an obtuse angle). Under waterlogging, all locations were negatively correlated (an obtuse angle). The presence of wide obtuse angles (i.e., strong negative correlations) among test environments is an indication of strong crossover GE. Under optimum conditions, there were obtuse angles (negative correlation) between Godhra and Kolhapur, Godhra and Ludhiana, Godhra and Hyderabad, Varanasi and Kolhapur, Varanasi and Hyderabad, Begusarai and Hyderabad, Begusarai and Kolahpur, and Ludhiana and Kolhapur. The angles between Godhra and Varanasi, Godhra and Begusarai, Varanasi and Begusarai, Varanasi and Ludhiana, Begusarai and Ludhiana, and Ludhiana and Hyderabad were acute; thus, they were positively correlated.

### 3.8 Plotting the heat map graphics for the BLUEs and BLUPs

**Figure 3:**
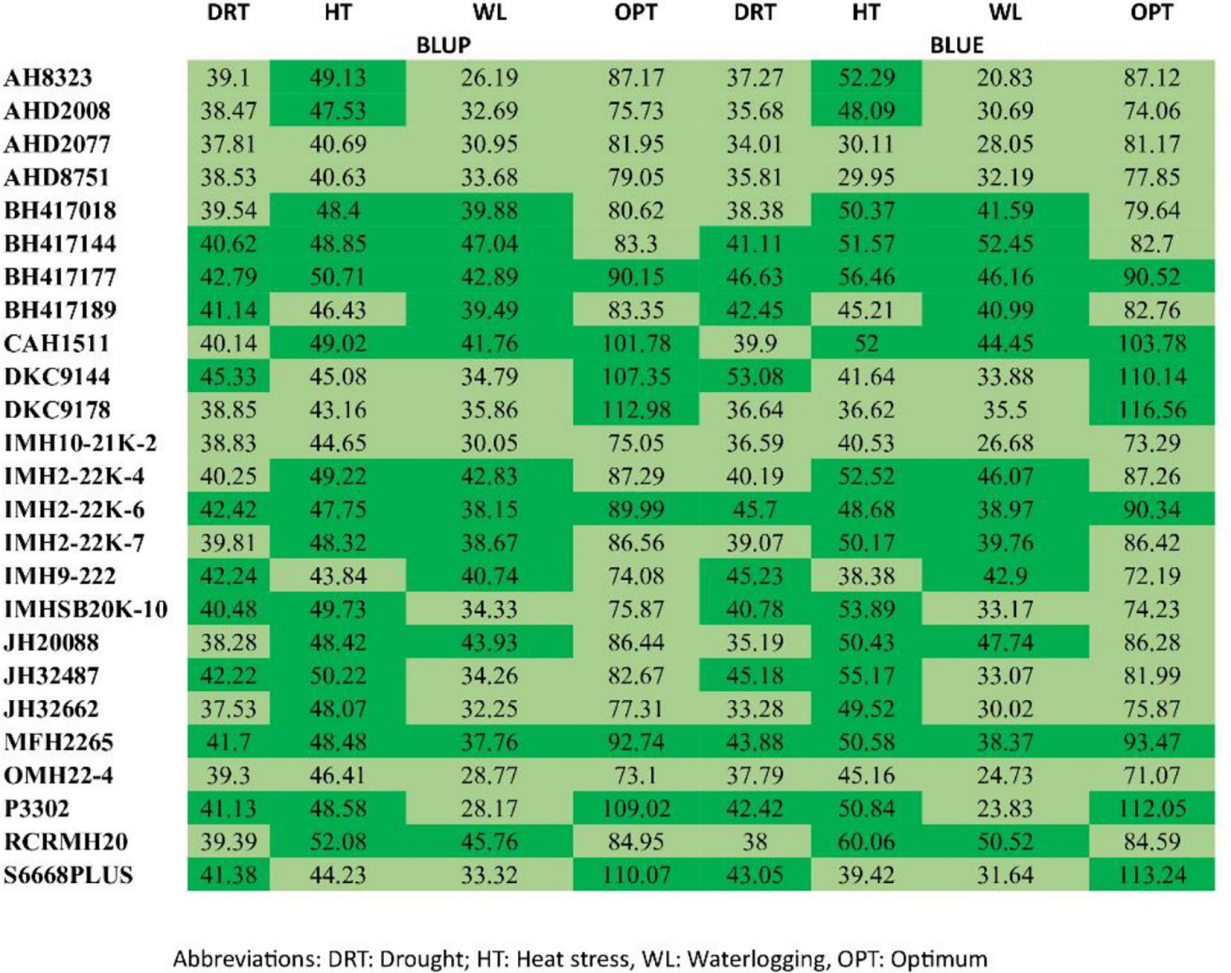
Ranks of genotypes illustrated by the BLUE and BLUP scores for grain yield.

For a more precise assessment of yield and stability, the experimental genotypes underwent ranking according to their BLUE and BLUP scores. **Figure 3** presents a heatmap that explores how different maize genotypes are ranked according to grain yield. An analysis of BLUEs and BLUPS scores revealed two distinct clusters of maize genotypes. The Light Green Cluster comprises genotypes characterized by both low productivity and instability, including AHD 2077, AHD 8751, IMH 10-21K-2, and OMH 22–4 across all the four different ecologies by both BLUEs and BLUPs. In contrast, the Dark Green Cluster comprises genotypes marked by both high productivity and stability, exemplified by BH 417144, MFH 2265, and BH 417177 across the ecologies, RCRMH 20 under HS and managed waterlogging.

### 3.9 Variance components and genetic parameters

Genotypic variance is the inherent variation, which remains unaltered by the environment. It is the variation due to the genotypes. Residual variances were higher than genotypic variance at all three distinct locations under drought (**Table 10**), whereas under HS, majorly genotypic variances were higher than the residual one. Similar results were observed for waterlogging conditions. As per optimum conditions, genotypic variances were higher at Godhra, Begusarai, and Hyderabad whereas residual variances were higher at Kolhapur, Varanasi, and Ludhiana.

**Table 10:** Variances and heritability under different locations within the diverse environments.

Environment	Location	Genotype variance	Residual variance	Heritability	Heritability (%)
Drought	Godhra	9.41	141.14	0.12	12.0
	Hyderabad	1.80	53.66	0.06	6.0
	Kolhapur	20.17	30.64	0.57	57.0
Heat	Begusarai	90.03	77.42	0.70	70.0
	Godhra	69.62	70.19	0.66	66.0
	Kolhapur	154.72	30.48	0.91	91.0
Waterlogging	Hyderabad	465.92	31.56	0.97	97.0
	Ludhiana	344.41	20.47	0.97	97.0
	Varanasi	458.31	9.50	0.99	99.0
Optimum	Begusarai	112.25	23.60	0.90	90.0
	Godhra	67.62	13.72	0.91	91.0
	Hyderabad	283.95	9.86	0.98	98.0
	Kolhapur	98.66	107.49	0.65	65.0
	Ludhiana	42.80	108.82	0.44	44.0
	Varanasi	37.20	180.45	0.29	29.0

Heritability measured the association between genotype and phenotype, estimated using a statistical parameter known as variance. Heritability plays an important role in the selection process in the plant breeding especially in the selection of elite genotypes in a segregating population. Heritability is categorized as low (<30%), medium (30%-60%), and high (>60%) (Johnson et al., 1955). Under drought ecology, heritability was low at Godhra (12%) as well as at Hyderabad (6%) and moderate at Kolhapur (57%). Under HS, there was high heritability at all three locations, i.e., Begusarai, Godhra, and Kolhapur (70%, 66%, and 91%). For waterlogging, high heritability was reported for all three tested locations (Hyderabad: 97%, Ludhiana: 97%, and Varanasi: 99%). Under optimum crop growth conditions, Begusarai, Godhra, Kolhapur, and Hyderabad had high heritability (90%, 91%, 98%, and 65%, respectively) whereas Ludhiana had medium (44%) and Varanasi had low (29%) heritability (**Table 10**).

To conclude, genotype RCRMH20 was identified as the best-performing genotype under heat and at water logging with mean yields of 60.07 q/ha (E1: 42.10, E2: 45.0, and E3: 93.00) and 50.52 q/ha (E1: 47.5, E2: 52.5, and E3: 51.4). BH 417177 had 46.6 q/ha (drought), 56.4 q/ha (heat), and 46.16 q/ha (waterlogging) mean grain yields. JH 32487 is an early maturity hybrid whereas both BH 417189 and BH 417177 have medium maturity. Early maturity also plays a pivotal role by allowing plants to complete their life cycle before the onset of water deficit period by drought escape. It helps to minimize the grain yield losses particularly under drought stress. Also, if the crop plants mature earlier, then it enables them to complete the critical growth stages (flowering and grain filling) before the hottest time of the season. Currently, many breeding programs actively incorporate earliness as a major trait for developing climate resilient crop varieties.

## 4 Discussion

Our comprehensive investigation by GGE biplot and linear mixed models provided information into the performance of 25 maize hybrids under different abiotic stresses. The genetic makeup of the genotypes, the environmental conditions of the locations, and their interactions had a significant impact on the yield and associating yield components of the different maize genotypes.

In the analysis of variance, the significant GEI effects (under heat stress and optimal growth conditions) observed in the present study indicates that the evaluated genotypes do not exhibit consistent performance across different test environments. This highlights the importance of investigating the nature and magnitude of GEI. The findings of this study align with previous research on how various environmental factors influence the performance of maize genotypes. In a study carried out by Kumar et al. (2023), in the evaluation trial of 30 maize hybrids in three diverse environments, viz., drought, rainfed and optimal conditions, environments, and genotypes, and Genotype × Environment interactions (G × E) were found to be highly significant.

BLUPs predict the breeding value or genetic potential of genotypes, treating them as random samples from a population with a known variance structure. In BLUE, the effect of a fixed factor (a variety or a treatment) within a specific trial or series of trials is estimated. In a study carried out by Oliveira et al. (2016), eight synthetic varieties of maize were sown in a diallel design of mating. The hybrids and their parents were then evaluated in three separate environments. Both combining ability and predicted breeding values (BLUPs) were estimated using PROC MIXED and PROC COR functions of SAS 9.3. Correlations between estimated GCA and SCA and BLUP values were moderate to high. Significant Genotype × Environment interactions (G × E) were observed. As per Bal et al. (2025), the evaluation of the hybrids across multienvironment trials under irrigated and non-irrigated conditions was done using BLUPs. Hybrid H3 demonstrated high yield stability across both irrigated and non-irrigated trials. The results of BLUP, AMMI, GGE, and WAASBY analyses consistently highlighted H3 as the top-performing hybrid, exhibiting high stability across both irrigated and non-irrigated conditions. In a study conducted by Kunwar et al. (2024), which used for statistical methods (both fixed effect and linear mixed models), i.e., genotype–environment interactions (AMMI), visually analyzing genotype performance and stability across environments (GGE), breeding values of genotypes for selection (BLUP), and multiple traits for selection (MTSI), their study revealed significant (P < 0.001) impacts of genotype, environment, and their interaction (GEI) on yield.

In maize, validation results have indicated that best linear unbiased predictions (BLUP) are useful, not only for routine selection of single crosses but also for choosing F2 populations for inbred development (Bernardo, 1999). The BLUP, although not yet widely used for annual plants, has shown great potential for more relevant genetic progress (Bernardo, 1996; Jesus Freitas et al., 2014). Employing variance component estimates by REML and predicting breeding values by BLUP can be efficiently used (Resende and Sturion, 2001). The breeder could therefore predict progeny values by focusing only on the most promising combinations. There is a series of publications (Parisseaux and Bernardo, 2004; Charcosset et al., 1998) on the prediction of hybrid performance in crosses of inbred lines from two different heterotic pools in maize (*Zea mays*) using the coefficient of co-ancestry to model general combining ability (GCA) and specific combining ability (SCA) effects. Cross-validation studies showed that BLUP provides good predictions. Reis et al. (2005) demonstrated both by simulation and by cross-validation with maize data that BLUP of hybrid performance had better accuracy than BLUE. According to Arnhold et al. (2009), it is better to use the mixed model methodology for breeding programs and BLUP is preferable to BLUE, as BLUP can be used to estimate the breeding values of individuals. More often, BLUP estimates are found to be more precise than BLUE derived through fixed effect models.

Together, the first and second principal components (PC1 and PC2) explained 100% of variation in drought (PC1: 50.0, PC2: 50.0), heat stress (PC1: 59.61, PC2: 40.39), and water logging (PC1: 94.23, PC2: 5.77) conditions as per GGE biplot analysis. Kumar et al. (2020) conducted a study to assess the effect of environment and yield stability of 68 quality protein maize hybrids at three test environments. Together, the two principal coordinates axes (PCA) explained 100% of phenotypic variation.

The overall locations mean that grain yields under water deficit, high temperature, waterlogging, and optimal irrigated conditions were 40.29, 47.19, 36.57, and 87.5 q/ha, respectively. Higher yields recorded under optimal conditions than under drought, high temperature, and waterlogging conditions highlight the significant effects of abiotic stress at the experimental sites. Drought stress has been recognized as a major constraint in maize production as it significantly reduces the grain yield by affecting the water relations of plants at cellular, tissue, and organ levels causing damage and adaptation reactions. In a study carried out by Bruce et al. (2002), an evaluation trial for maize hybrids was planted in Weslaco, Texas, in 2000, which recorded mean grain yields of 5.1 and 3.0 t/ha, under optimum and water deficit conditions, respectively, which resulted in approximately 40% yield losses. Water logging caused by contingent flooding and continuous rainfall coupled with inadequate drainage or a high water table is another important limitation (Zaidi et al., 2010). In maize, this anaerobic condition leads to the unbalanced transport of both nutrients and water to the leaf tissue, reduced leaf water potential, stomatal closure, leaf curling (Sairam et al., 2008), and yield reduction (Ren et al., 2014).

As described by Mano and Omori (2007), three primary physiological mechanisms conditioning water logging tolerance are the capacity to form root aerenchyma, the ability to grow adventitious/brace roots at the soil surface during flooding conditions, and tolerance to toxins (e.g., Fe^2+^ and H_2_S) under reduced soil conditions. Understanding the genetics of such physiological traits can aid in more precise manipulation of waterlogging tolerance in the maize breeding programs. As per Mano et al. (2005), the adventitious root-forming (ARF) ability of the plants at the soil surface is most important in the adaptation of the plant to flooding or waterlogging conditions. The ARF ability of maize and teosinte seedlings under waterlogged conditions was evaluated. The ARF ability was evaluated by visual rating of root formation at the soil surface after exposure to flooding for 2 weeks. The ARF ability varied among 43 maize and teosinte lines, with teosinte showing a higher ARF ability as compared with cultivated maize.

In the present study, results indicated that tested hybrids RCRMH 20, BH 417144, MFH 2265, and BH 417177 emerged as the most stable hybrids. MFH 2265 was contributed by Tirhut College of Agriculture, Dholi whereas BH 417144 and BH 417177 are under Maize Research Centre, PJTSAU. Genotype RCRMH 20 (contributed by University of Agricultural Sciences, Raichur) was identified as the best performing genotype under heat and at water logging with mean yields of 60.07 and 50.52 q/ha, respectively. These genotypes have demonstrated exceptional performance in adverse climates, which may indicate that parents of these hybrids may be used for further breeding specifically for stress environments. Selected hybrids across the abiotic stresses, which can grow in any adverse conditions under subtropical conditions, can play an important role for food security. The usage of BLUP and BLUE rather than conventional ANOVA-based statistical methods is another novelty of the study along with correlations between locations and principal components.

## 5 Conclusion

The GGE biplot and LMM assess and understand genotype–environment interactions, helping researchers interpret and use trial data for further improvement in breeding programs. The stable as well as high-yielding genotypes identified in this study could be tested in larger plot size at multi-locations so that they may be recommended for commercial cultivation suited for an appropriate environment. Their genetic potential can be further exploited by testing at multi-locations. This study has important implications in determining the appropriate test location for development of cultivars. The most stable genotypes (RCRMH 20, BH 417144, MFH 2265, and BH 4171770) identified under different abiotic stress selection trials can be further exploited for generating information on differential performance. Maize is such a substantial crop for both food and feed purposes in the world, and there is tremendous interest in and demand for improving maize drought tolerance. Moreover, to improve productivity of rainfed maize also, the development of hybrids with tolerance to drought stress is an important objective in maize breeding programs.

## Data Availability

The original contributions presented in the study are included in the article/**Supplementary Material**. Further inquiries can be directed to the corresponding author.

## Supplementary material

Supplementary Figure 1 **(A)** Agrometerological data (rainfall, mean temperature and relative humidity) for Godhra location under drought and heat stress conditions. **(B)** Agrometerological data (rainfall, mean temperature and relative humidity) for Kolhapur location under drought and heat stress conditions. **(C)** Agrometerological data (rainfall, mean temperature and relative humidity) for Hyderabad location under drought and waterlogging conditions.

Supplementary Figure 2 **(A)** Agrometerological data (rainfall, mean temperature and relative humidity) for Begusarai location under heat stress conditions.

Supplementary Figure 3 **(A)** Agrometerological data (rainfall, mean temperature and relative humidity) for Ludhiana location under waterlogging conditions. **(B)** Agrometerological data (rainfall, mean temperature and relative humidity) for Varanasi location under waterlogging conditions.
