# Supplementary material for: Stress-resilient maize hybrids with stable agronomic performance: a pursuit to strengthen sustainable maize production in India
Source: Front Plant Sci. 2025 Nov 26;16:1714259. doi: 10.3389/fpls.2025.1714259 (PMC12689321; doi:10.3389/fpls.2025.1714259)
Supplement: Supplementary file 1 [file SupplementaryFile1.docx]

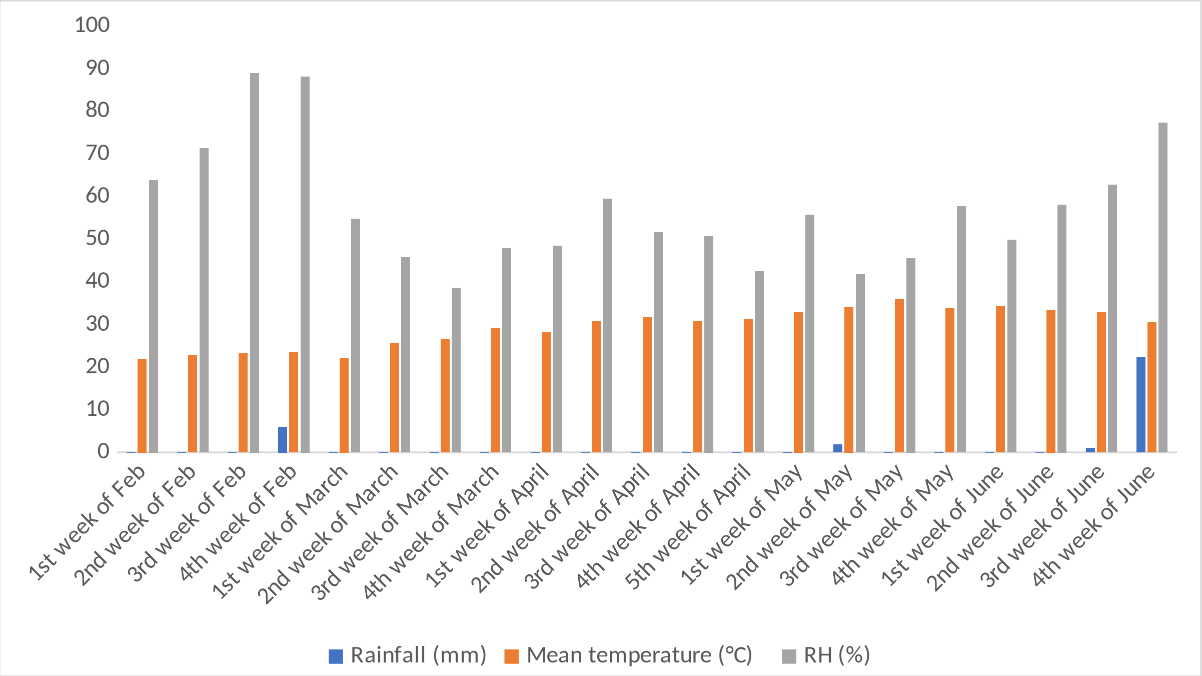
100
90
80
70
60
50
40
30
20
10
0
1st week of Feb
2nd week of Feb
3rd week of Feb
4th week of Feb
1st week of March
2nd week of March
3rd week of March
4th week of March
1st week of April
2nd week of April
3rd week of April
4th week of April
5th week of April
1st week of May
2nd week of May
3rd week of May
4th week of May
1st week of June
2nd week of June
3rd week of June
4th week of June
Rainfall (mm)
Mean temperature (°C)
RH (%)

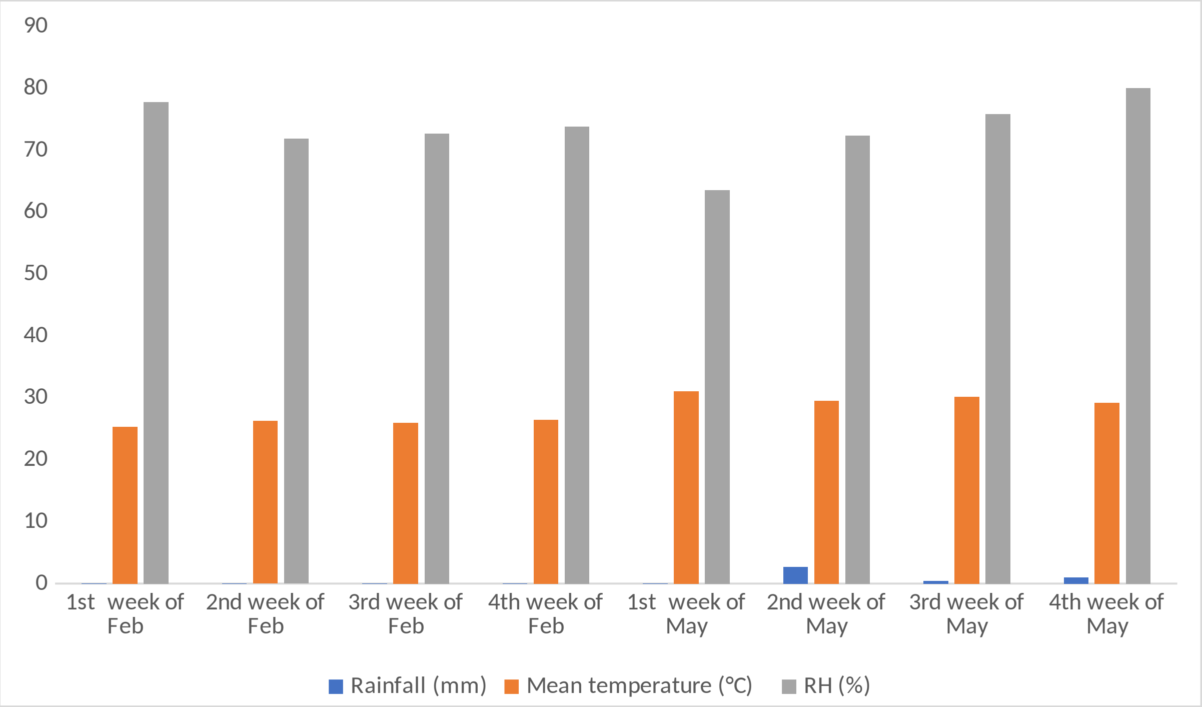
90
80
70
60
50
40
30
20
10
0
1st week of Feb
2nd week of Feb
3rd week of Feb
4th week of Feb
1st week of May
2nd week of May
3rd week of May
4th week of May
Rainfall (mm)
Mean temperature (°C)
RH (%)

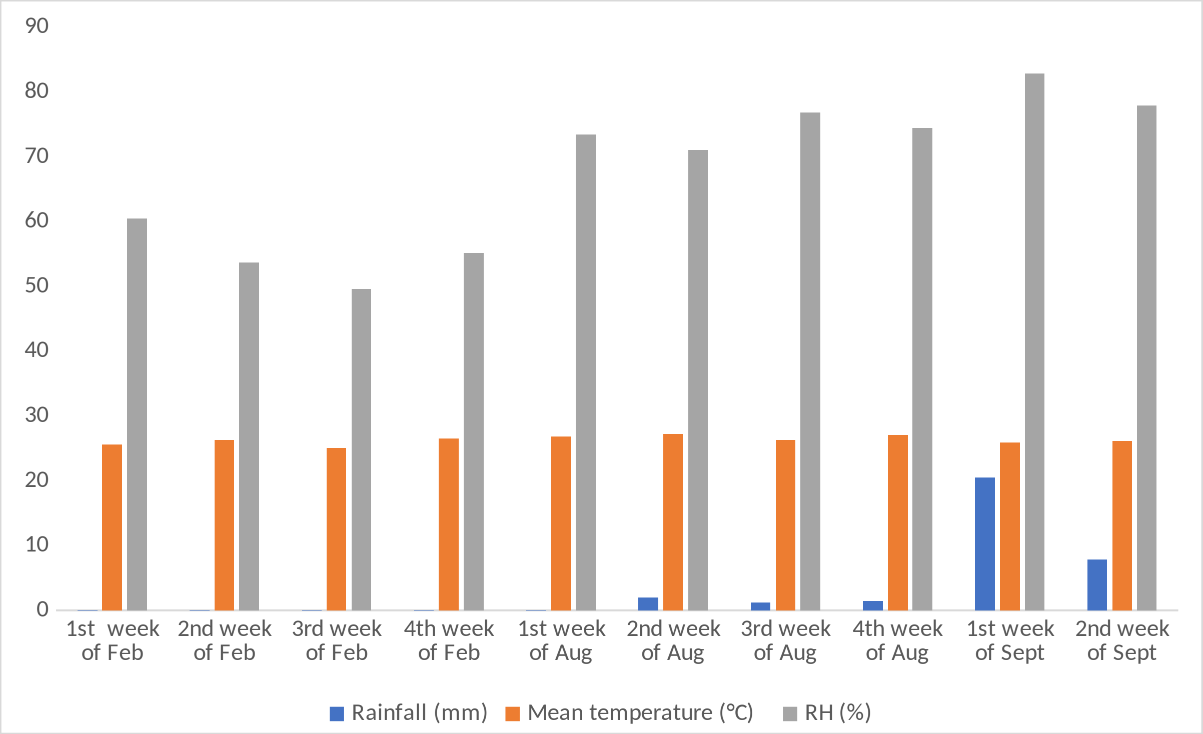
90
80
70
60
50
40
30
20
10
0
1st week of Feb
2nd week of Feb
3rd week of Feb
4th week of Feb
1st week of Aug
2nd week of Aug
3rd week of Aug
4th week of Aug
1st week of Sept
2nd week of Sept
Rainfall (mm)
Mean temperature (°C)
RH (%)

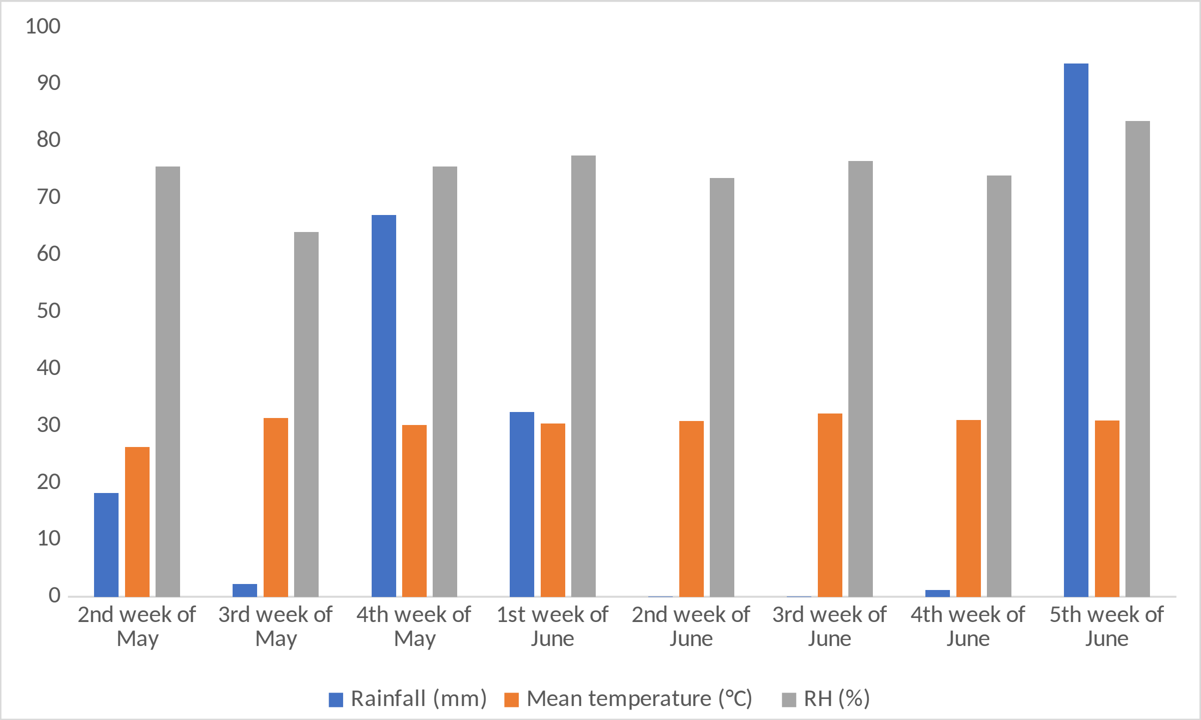
100
90
80
70
60
50
40
30
20
10
0
2nd week of May
3rd week of May
4th week of May
1st week of June
2nd week of June
3rd week of June
4th week of June
5th week of June
Rainfall (mm)
Mean temperature (°C)
RH (%)

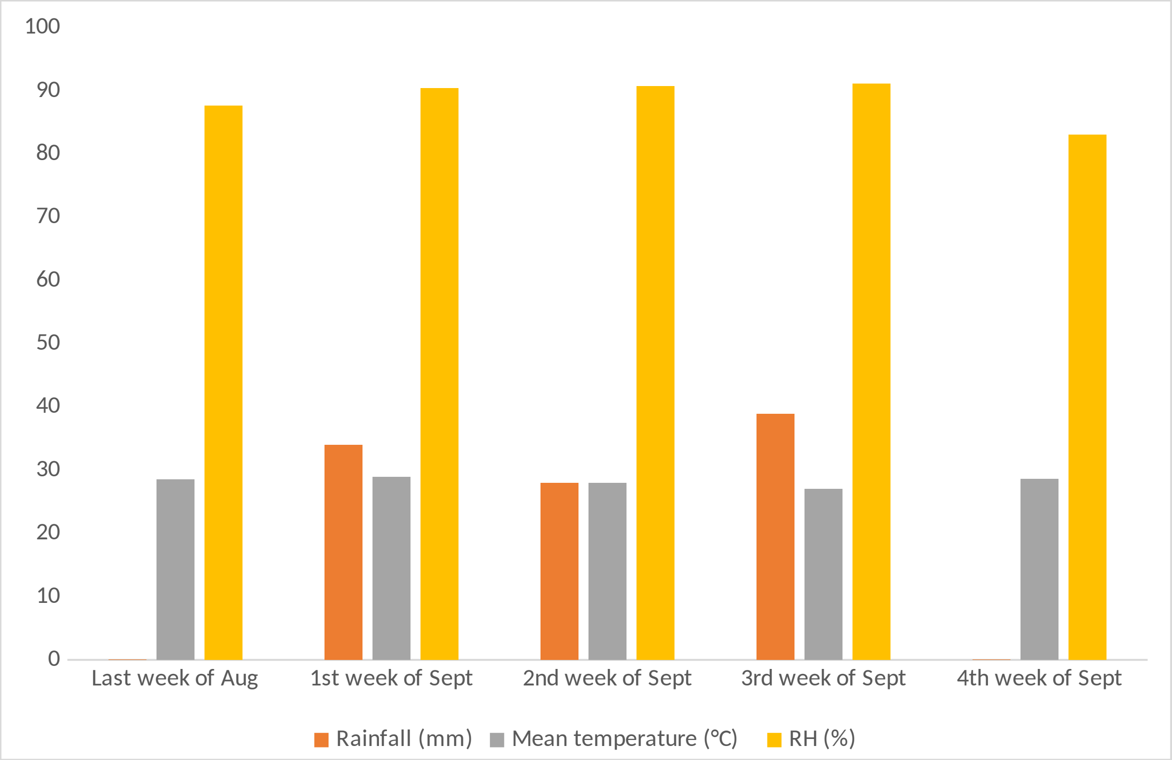
100
90
80
70
60
50
40
30
20
10
0
Last week of Aug
1st week of Sept
2nd week of Sept
3rd week of Sept
4th week of Sept
Rainfall (mm)
Mean temperature (°C)
RH (%)

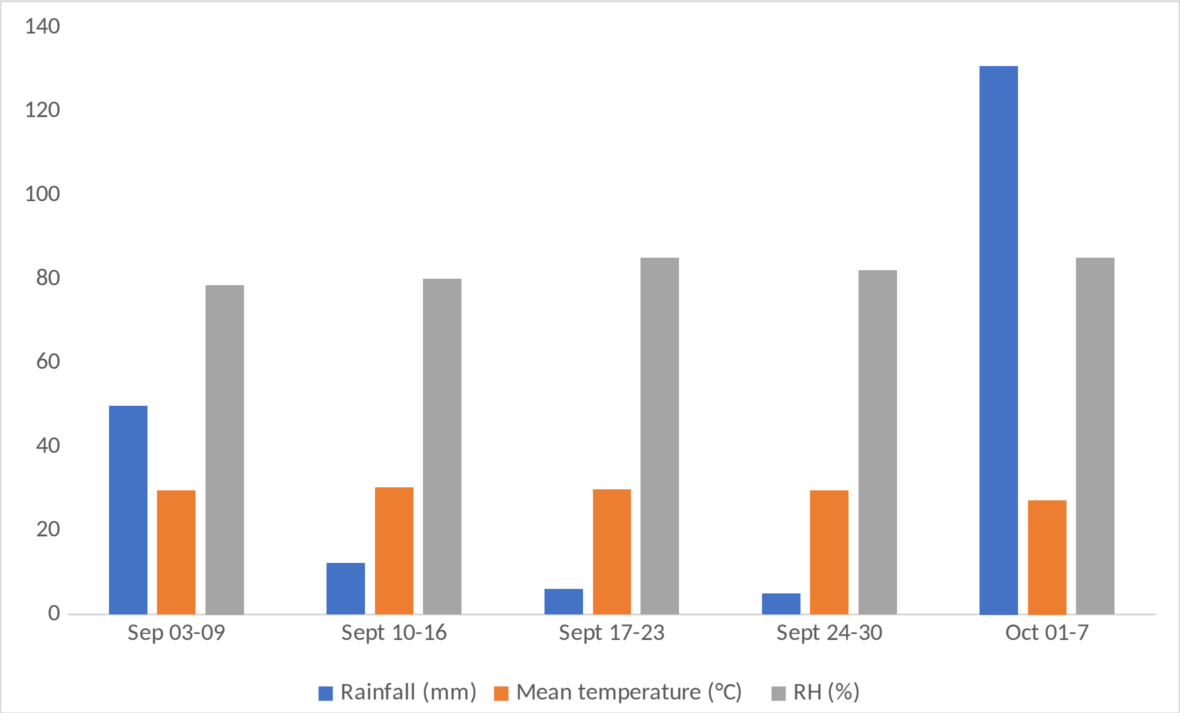
140
120
100
80
60
40
20
0
Sep 03-09
Sept 10-16
Sept 17-23
Sept 24-30
Oct 01-7
Rainfall (mm)
Mean temperature (°C)
RH (%)
